# Autologous, allogeneic, induced pluripotent stem cell or a combination stem cell therapy? Where are we headed in cartilage repair and why: a concise review

**DOI:** 10.1186/s13287-015-0086-1

**Published:** 2015-05-15

**Authors:** Lucienne A. Vonk, Tommy S. de Windt, Ineke C. M. Slaper-Cortenbach, Daniël B. F. Saris

**Affiliations:** Department of Orthopaedics, University Medical Center Utrecht, HP G05.228, PO Box 85090, 3508 GA Utrecht, The Netherlands; Cell Therapy Facility, Department of Clinical Pharmacy, University Medical Center Utrecht, HP F03.821, PO Box 85090, 3508 GA Utrecht, The Netherlands; MIRA institute, University Twente, ME125, PO Box 217, 7500 AE Enschede, The Netherlands

## Abstract

The evolution of articular cartilage repair procedures has resulted in a variety of cell-based therapies that use both autologous and allogeneic mesenchymal stromal cells (MSCs). As these cells are increasingly available and show promising results both *in vitro* and *in vivo*, cell-based strategies, which aim to improve ease of use and cost-effectiveness, are progressively explored. The use of MSCs in cartilage repair makes it possible to develop single-stage cell-based therapies. However, true single-stage procedures rely on one intervention, which will limit cell sources to fraction concentrates containing autologous MSCs or culture-expanded allogeneic MSCs. So far, it seems both autologous and allogeneic cells can safely be applied, but clinical studies are still ongoing and little information on clinical outcome is available. Further development of cell-based therapies may lead to clinical-grade, standardized, off-the-shelf products with easy handling for orthopedic surgeons. Although as of yet no preclinical or clinical studies are ongoing which explore the use of induced pluripotent stem cells for cartilage repair, a good manufacturing practice-grade induced pluripotent stem cell line might become the basis for such a product in the future, providing that cell fate can be controlled. The use of stem cells in clinical trials brings along new ethical issues, such as proper controls and selecting primary outcome measures. More clinical trials are needed to estimate detailed risk-benefit ratios and trials must be carefully designed to minimize risks and burdens for patients while choosing outcome measures that allow for adequate comparison with results from similar trials. In this review, we discuss the different aspects of new stem cell-based treatments, including safety and ethical issues, as well as provide an overview of current clinical trials exploring these approaches and future perspectives.

## Introduction

Cartilage defects in the weight-bearing joint are a severe limitation to the patient and pose a significant burden to society. Symptoms include pain, stiffness, joint effusion and locking, which cause considerable disability and decrease quality of life. It is well understood that cartilage defects need (early) treatment because they have a poor intrinsic healing capacity and tend to lead to osteoarthritis [1].

Cartilage repair strategies have rapidly evolved over time; in 1950 the resection of loose and damaged tissue was the only treatment available. In the late 1980s microfracture was introduced, which involves drilling multiple holes in the subchondral bone to allow an influx of bone marrow that stimulates natural repair. In 1994, the first results on autologous chondrocyte implantation (ACI) were published [2] and many generations of cell therapy have followed [3]. In first generation ACI, chondrocytes isolated from a biopsy of a non-weight bearing location in the knee were culture-expanded and subsequently implanted under a periosteal cover. In the second generation, a cover of collagen or a resorbable biofilm replaced the periosteal cover. Next, open collagen cell carriers were introduced, which led to the manufacture of bioactive matrices to improve hyaline cartilage formation. Currently, matrix-based arthroscopic application and advanced delivery through bio-airbrush technology are being applied. Much attention was also given to the culture-expansion phase resulting in the introduction of characterized cells that show the most chondrogenic potential and establishing release criteria and production guidelines.

**Fig. 1:**
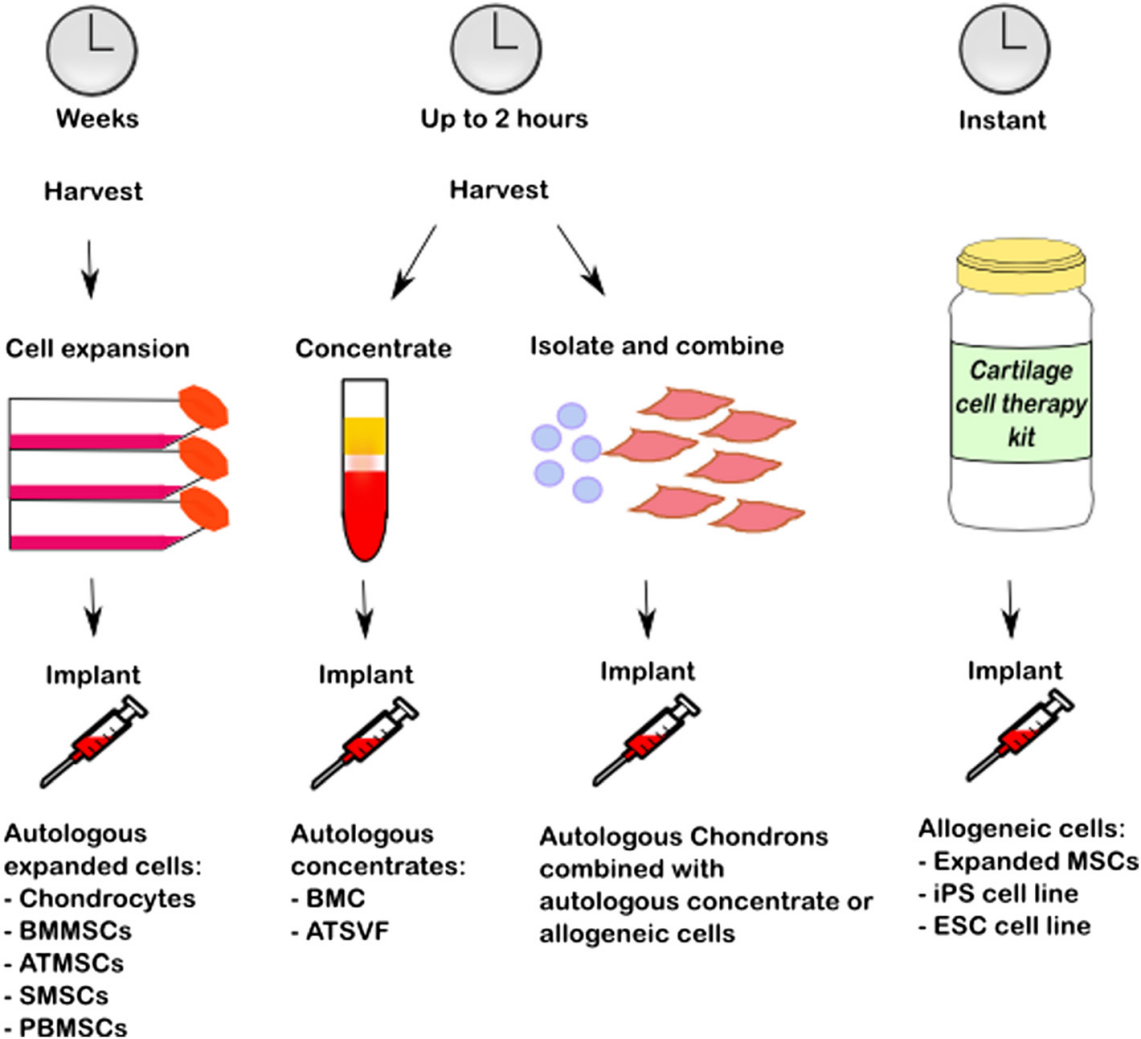
Cell-based therapies for cartilage defects have evolved through a few generations with various cell sources. Using expanded autologous cells, one cell type can be used, but the cell expansion can take several weeks. Traditionally, autologous chondrocytes were used, but autologous mesenchymal stromal cells (MSCs) derived from several sources, such as bone marrow (BM) adipose tissue (AT), synovium (S) and peripheral blood (PB) are increasingly used. A single-stage cell-based treatment relies on obtaining sufficient cells within the time frame of a single surgery. Options being explored are autologous MSC-rich concentrates, such as a bone marrow concentrate (BMC), or the vascular stromal fraction from adipose tissue (ATVSF) or a combination of rapidly isolated autologous chondrons combined with allogeneic MSCs or an autologous MSC-rich concentrate. An off-the-shelf product that is readily available could consist of expanded allogeneic MSCs or an induced pluripotent stem (iPS) cell line or an embryonic stem cell (ESC) line. ATMSC, adipose tissue-derived mesenchymal stromal cell; BMMSC, bone marrow-derived mesenchymal stromal cell; PBMSC, peripheral blood-derived mesenchymal stromal cell; SMSC, synovium-derived mesenchymal stromal cell

The mid- to long-term results of ACI have been encouraging [2,3]. However, the limitations of this extensive procedure in terms of patient burden and costs have steered cartilage repair towards single-stage procedures and off-the-shelf cellular or biomaterial-based products. The challenge for a single-stage approach lies in obtaining sufficient cells. Due to the low cell number in native cartilage and the large surface area to volume ratio of cartilage defects, it is impossible to obtain sufficient autologous chondrocytes without expanding them. Therefore, the answer could lie in supplementing or replacing them with multipotent mesenchymal stem or stromal cells (MSCs; Fig. 1). However, the fate of MSCs *in vivo* remains unknown: will they survive or disappear in the long-term? Will they all differentiate into chondrocytes or will some remain as MSCs? Current studies are not conclusive on these questions; some have suggested MSCs differentiate and survive *in vivo* up to 6 months, while others suggest MSCs have a chondroinductive role - that is, stimulate cartilage regeneration through trophic factors while slowly disappearing from the culture [4]. Although it remains unclear what the exact fate of these MSCs will be *in vivo*, MSCs of both autologous and allogeneic origin have increasingly been introduced for cartilage repair in clinical studies.

The development of an off-the-shelf product to treat cartilage defects would rely on autologous MSC-rich concentrates, allogeneic MSCs or induced pluripotent stem cell (iPSC) lines. However, rapid developments in the field make it difficult to evaluate the existing evidence for such cellular therapies in terms of preclinical and clinical safety and early efficacy. The purpose of this review is to provide a concise overview of the available literature on autologous and allogeneic MSCs for cartilage repair of focal defects. Besides clinical studies, the sources of MSCs, safety and ethical issues with respect to allogeneic MSCs, the use of iPSCs and future perspectives are discussed.

## Sources for mesenchymal stromal cells

Minimal criteria to define expanded multipotent human MSCs, as defined by the International Society for Cellular Therapy, include that they must be plastic-adherent when maintained in standard culture conditions, express CD105, CD73 and CD90, and lack expression of CD45, CD34, CD14 or CD11b, CD79α or CD19 and HLA-DR surface molecules, and they must be capable of differentiating into osteoblasts, adipocytes and chondroblasts *in vitro* [5]. MSCs can be isolated and expanded from a variety of sources, such as bone marrow, adipose tissue, synovial membrane, synovial fluid, umbilical cord blood, peripheral blood, dermis, trabecular bone, infrapatellar fat pad, dermis, periosteum and muscle. The phenotypic characteristics of MSCs derived from different sources are similar, but the number of MSCs and their proliferation and differentiation potentials can differ [6]. Bone marrow is often used as a source for MSCs (BMMSCs). Although only a small percentage of its mononuclear fraction consists of BMMSCs, they are relatively easy to isolate and expand and they have a high potential for differentiation [7]. The stromal vascular fraction of adipose tissue contains more MSCs (ATMSCs) compared with bone marrow (as measured in a colony-forming unit-fibroblasts (CFU-F) assay) and harvesting adipose tissue is less invasive [8]. ATMSCs show enhanced rates of proliferation and they can undergo more population doublings before senescence [8,9]. However, the *in vitro* chondrogenic potential of ATMSCs is lower compared with BMMSCs *in vitro*, especially when pellet cultures are stimulated with transforming growth factor (TGF)-beta. [9]. The tissue formed by ATMSCs chondrogenically differentiated with TGF-beta contained less type II collagen and proteoglycans compared with tissue formed by chondrogenically differentiated BMMSCs from the same donors. The exact reason is unknown, but it is suggested there might be less chondroprogenitor cells present in the ATMSC population or that the expansion favors clonal expansion of cells with higher proliferative rates, albeit with less differentiation potential [9]. However, other studies have shown a good chondrogenic potential of ATMSCs when bone morphogenetic protein (BMP)-6 was used, which may be explained by an altered TGF-beta receptor and BMP profile of ATMSCs compared with BMMSCs [10,11].

MSCs derived from the synovial membrane (SMSCs) can be harvested through an arthroscopic procedure or from synovial fluid. The amount of SMSCs in synovial fluid is very low; only about 14 cells per milliliter of synovial fluid from healthy donors can form CFU-F colonies. Parts of these colony-forming cells are considered SMSCs as they can differentiate into the adipogenic, osteogenic and chondrogenic lineages. Compared with BMMSCs and ATMSCs, they have a higher rate of proliferation [12,13]. Sakaguchi and colleagues showed superior chondrogenic differentiation of SMSCs compared with donor-matched BMMSCs, ATMSCs and MSCs from periosteum and skeletal muscle *in vitro* [14]. SMSCs have also shown potential in *in vitro* generation of hyaline cartilage tissue-engineered constructs [15]. Implantation of these *in vitro*-generated constructs showed good repair of cartilage defects in a pig model with SMSCs isolated from both immature and mature pigs [16,17].

MSCs can also be isolated from peripheral blood (PBMSCs) [18]. MSC isolation from blood provides low cell numbers, but peripheral blood can be easily obtained in a non-invasive way. Although there is a large variation in the success rates of isolation of MSCs from umbilical cord blood (UMSCs), they have good chondrogenic potential [19]. The accessibility of UMSCs along with their efficient expanding characteristics have made allogeneic UMSCs the only off-the-shelf cell product for cartilage repair [20]. MSCs can also be isolated from the periosteum, but the limited availability and complex tissue harvest procedure forms a barrier for their use. Currently, isolated BMMSCs and bone marrow concentrates (BMCs) are most commonly used for treatment of cartilage defects in a clinical trial setting (Table 1).

**Table 1:** Overview of clinical studies applying autologous mesenchymal stromal cells to a cartilage defect

Cell type	Study type	Number of patients	Follow-up	Results	Reference
BMMSC	Case report	2	5 years	Clinical improvement and defect fill with fibrocartilage	[25]
BMMSC	Case report	1	1 year	Bone and cartilage repair	[26]
BMMSC	Case report	3	17-27 months	Clinical improvement and defect fill with fibrocartilage	[27]
BMMSC	Case report	1	12 months	Clinical improvement and defect fill with hyaline tissue	[28]
BMMSC	Case series	5	12 months	Clinical improvement and defect fill	[29]
BMMSC	Case report	2	31 months	Clinical improvement and defect fill	[30]
BMC	Case series	48	4 years	Clinical improvement and defect fill	[31,32]
BMC	Case series	20	24 months	Bone and cartilage repair	[33]
BMC	Case series	5	12 months	Defect fill with hyaline to fibrocartilaginous tissue	[34]
BMC	Case series	54	5 years	Clinical improvement and good integration of repair tissue	[35]
BMC	Case series	15	2 years	Clinical improvement and defect fill with hyaline tissue	[36]
BMC and ACy	Case series	40	Interim results 1 year	Clinical improvement and defect fill with hyaline tissue	[37] NCT01041885
SMSC					[16,17]
BMMSC	Comparative study	36 (total 72)	24 months	Clinical improvement, defect fill with hyaline tissue	[38]
BMC	Comparative study	25 (total 81)	36 months	Clinical improvement and defect fill with hyaline tissue	[39]
PBMSC or BMC	Comparative study	25 PBMSC, 21 BMC	5 years	Clinical improvement and defect fill for both groups	[40]
BMMSC	Case series	25	12 months		NCT00891501
BMMSC	Case series	6	12 months		NCT00850187
BMC	Case series	140	36 months		NCT02005861
BMC	Case series	50	12 months		NCT01159899
ATSVF	Comparative study	40	24 months		NCT02090140
ATMSC	Comparative study	30	18 months		NCT01399749
BMMSC	Comparative study	50 in total	5 years		NCT00885729

*ACy* autologous chondrocyte, *ATMSC* adipose tissue-derived mesenchymal stromal cell, *ATSVF* adipose tissue stromal vascular fraction, *BMC* bone marrow concentrate, *BMMSC* bone marrow-derived mesenchymal stromal cell, *PBMSC* peripheral blood-derived mesenchymal stromal cell, *SMSC* synovium-derived mesenchymal stromal cell

One of the concerns with using MSCs for cartilage repair is that if they do differentiate into the chondrogenic lineage and engraft the new cartilage, they might undergo terminal differentiation and become hypertrophic, as the default route of chondrogenic differentiation is terminal differentiation [21]. This concern is not limited to MSCs only, as chondrocytes can also undergo hypertrophic differentiation, which has been found in ACI [22].

Articular cartilage itself, especially the superficial layers, is also a reservoir for progenitor cells with multilineage potential [23,24]. Cartilage-derived progenitors even have a decreased potential for osteogenic and hypertrophic differentiation. Although the research on cartilage-derived progenitor cells is still very limited, a goat study has proven their ability to repair chondral defects. Williams and colleagues [24] suggested that about 0.7% of all cells in cartilage are progenitor cells.

## Clinical studies using autologous mesenchymal stromal cells

Since Wakitani and colleagues [25] performed the first treatment of full-thickness cartilage defects with autologous MSCs in 2004, autologous MSCs and MSC-rich concentrates are increasingly used for cartilage repair (overview provided in Table 1). Most published results are obtained from low level (IV or V) evidence studies [25-37], and few comparative studies are available [38-40]. Nejadnik and colleagues [38] compared the implantation of BMMSCs (36 patients) with first-generation ACI (36 matched patients) in a cohort study (evidence level III). Based on clinical and subjective improvement up to 2 years postoperatively, it was concluded that BMMSCs are as effective as chondrocytes for articular cartilage repair. Histological evaluation of biopsies taken from a few patients (four BMMSC, three ACI) showed hyaline-like cartilage tissue and no abnormal calcification or necrosis. Interestingly, patients younger than 45 years scored better than patients aged over 45 years in the ACI group, while age did not make a difference in the BMMSC group. Following several case series, Giannini and colleagues [31-33] reported on a one-step approach to treat osteochondral talar dome defects and compared a MSC-rich BMC (25 patients) with ACI (10 patients) and an arthroscopic ACI (46 patients) (evidence level IV) [39]. As in the previously described study, similar clinical improvement was observed, and magnetic resonance imaging (MRI) and histological evaluation showed complete defect fill with hyaline-like cartilage tissue in the majority of patients.

Only one study compared the use of two MSC-based treatments for cartilage repair [40]. In this study, 21 patients were treated with BMCs and 25 with PBMSCs. Clinical improvement was found in a total of 40 patients, in which the patients treated with PBMSCs showed superior results compared with the patients treated with BMCs. Poor results were found for four patients in the BMC group and two patients in the PBMSC group. Although MRI was also performed in this study, no MRI results were reported.

Although only two studies directly compared MSC-based treatments with ACI [38,39], the conclusions from these studies do suggest that MSCs are a promising cell source for cartilage repair. This is supported by the findings in the level IV and V evidence studies that used BMMSCs or BMC for cartilage repair; all have reported clinical improvement with a follow-up period ranging from 1 year to 5 years [25,27-32,35,36,38-40]. The studies that included MRI analysis in their outcome measures reported complete defect fill [27-29,31-33,35,36] and mostly congruity with the native cartilage [29,35]. Histological evaluation of biopsies showed the reparative tissue was hyaline-like cartilage [28,33,35,36,38,39], fibrocartilage [25,27,31,32], or a mixture of both [26,34].

Several other studies using autologous MSCs or concentrates are still ongoing, including two studies using ATMSCs to treat cartilage defects (Table 1; NCT01399749 and NCT02090140). So far, ATMSCs have only entered the preclinical phase in cartilage repair. In clinical use, concentrated ATMSCs have been injected intra-articularly for treatment of osteoarthritis [41,42]. SMSCs have been used in preclinical studies, which gave promising results [16,17]. The tissue-engineered construct made by SMSCs as described in those preclinical studies is currently being explored in an investigator-driven phase I/II clinical trial in a small cohort in Japan.

Thus, only clinical results using expanded undifferentiated BMMSCs, PBMSCs or BMCs (bone-marrow derived buffy coat or the mononuclear fraction of bone marrow) are reported. Pre-differentiated MSCs have not been used as yet. Although MSCs and MSC-rich concentrates are promising for cartilage repair, a lack of comparative studies confines a prediction to what the optimal cell source for MSC-based cartilage repair would be. Moreover, MSCs and BMCs have been implanted using various cell carriers, passages and doses (sometimes even not reported; Tables 2 and 3), so much remains to be investigated and learned.

**Table 2:** Details on mesenchymal stromal cells used in clinical studies

Cell type	Cell carrier	Cell dose	Expansion medium	Passage	Characterization	Reference
BMMSC	Collagen gel	5 × 10^6^/ml	αMEM, 15% AS	Single passaged	CD73, CD90, CD105+, CD14, CD34, HLA-DR-	[25,27,28]
BMMSC	Hydroxyapatite ceramic	1.15 × 10^6^ in total	DMEM, 15% AS	Single passaged		[26]
BMMSC	Fibrin glue with periosteum	2 × 10^6^ cells/cm^2^	DMEM, 10% FBS, 50 μg/ml AA2P, 1% antibiotic-antimycotic	Single passaged	CD90, CD105+, CD14, CD34-	[38]
BMMSC	Platelet-rich fibrin gel	2 × 10^6^ cells/cm^2^	DMEM, 10% FBS, 1% pen/strep	Single passaged	CD73+, CD34, CD45-	[29]
BMMSC	Collagen scaffold		DMEM, 10%, FBS 1% pen/strep	Passaged		[30]
BMMSC	Membrane					NCT00885729
BMMSC	Periosteum			Passaged		NCT00891501
BMMSC	Type I collagen scaffold			Passaged		NCT00850187
PBMSC	Collagen membrane	1.25-5.2 × 10^6^	N/A	Unpassaged		[40]
ATMSC	Periosteum			Passaged		NCT01399749
SMSC	SMSC TEC					[16,17]
UMSC	Sodium hyaluronate			Passaged		[20] NCT01041001
BMMSC and AC	Fibrin glue	2 × 10^6^ cells/cm^2^	αMEM, 5% platelet lysate	Passage 3	CD73, CD90, CD105+, CD45, CD34, CD11b, CD14, CD31, CD79, CD19, HLA-DR-	[3,46] NCT02037204

*αMEM* alpha minimal essential medium, *AA2P* L-ascorbic acid 2-phosphate sesquimagnesium salt hydrate, *AC* autologous chondrons, *AS* autologous serum, *ATMSC* adipose tissue-derived mesenchymal stromal cell, *BMMSC* bone marrow-derived mesenchymal stromal cell, *DMEM* Dulbecco’s modified Eagle medium, *FBS* fetal bovine serum, *N/A* not applicable, *PBMSC* peripheral blood-derived mesenchymal stromal cell, *Pen/strep* penicillin/streptomycin, *SMSC* synovium-derived mesenchymal stromal cell, *TEC* tissue engineered construct, *UMSC* umbilical cord blood-derived mesenchymal stromal cell

**Table 3:** Details on bone marrow concentrates used in clinical studies

Type	Cell carrier	Harvest location	Amount harvested	Amount used	Reference
BMC	Collagen membrane	Ilium	27 ml	0.5-2.7 × 10^6^ cells	[40]
BMC	Collagen powder or hyaluronic acid membrane and platelet gel	Posterior iliac crest	60 ml	2 ml 10 × concentrated BM	[31-33,39] NCT02005861
BMC	Type I collagen scaffold	Iliac crest	60 ml		[34]
BMC	Collagen membrane	Ipsilateral iliac crest	60 ml	4-6 × concentrated BM, CFU-F/ml 2,000-5,700	[35]
BMC	Collagen I/III membrane	Ilium	30 ml		[36]
BMC	Protein matrix in collagen hydroxyapatite scaffold				NCT01159899
BMC and ACy	INSTRUCT scaffold				[37] NCT01041885
ATSVF	Collagen scaffold	Infrapatellar fat pad	5 cc		NCT02090140

*ACy* autologous chondrocyte, *ATSVF* adipose tissue stromal vascular fraction, *BM* bone marrow, *BMC* bone marrow concentrate, *CFU-F* colony-forming unit-fibroblasts

## Safety considerations using allogeneic mesenchymal stromal cells

It took until 2010 before the first clinical study exploring the use of allogeneic MSCs for cartilage repair started [20], probably due to the unknown risk of an immune response to allogeneic cells. MSCs have been shown to have low immunogenicity based on the lack of expression of markers such as CD45 and CD34 and HLA-DR surface molecules [43]. In addition, they are known to interact with immune cell populations and modulate the host immune responses [43]. Because of the immunosuppressive properties of MSCs, allogeneic MSCs are currently infused intravenously for the treatment of steroid-resistant graft-versus-host disease, acute respiratory distress syndrome and Crohn’s disease in clinical trials. However, as it remains unclear what the exact fate of these MSCs is *in vivo*, it cannot be excluded that the MSCs differentiate, leading to a loss in their immune-modulating property and a change in their immunogenicity [44]. Several preclinical studies in rabbits, pigs and goats showed effective cartilage repair after implantation of allogeneic MSCs in cartilage defects without any adverse events or rejection [17,45,46]. Moreover, no adverse events were reported when fully differentiated allogeneic chondrocytes or allogeneic cartilage pieces were transplanted in several animal and human clinical trials [47,48], possibly due to the immune privileged character of cartilage as it is avascular and has no lymphatic system. It has to be noted that cartilage defects are often debrided, which can cause penetration of the subchondral bone, allowing an influx of bone marrow. This might become a concern for using pre-differentiated allogeneic cells or allogeneic iPSCs.

## Clinical studies using allogeneic mesenchymal stromal cells

Only a few clinical trials have been initiated using allogeneic MSCs for cartilage repair (Table 4). In Korea, a phase III clinical trial comparing allogeneic UMSCs with sodium hyaluronate (CARTISTEM®, MEDIPOST, Korea) to microfracture treatment has recently finished. About 100 patients with articular cartilage defects were included in this study to assess the safety and efficacy with a follow-up of 48 weeks (NCT01041001). The safety of using allogeneic UMSCs was confirmed and histological analyses showed repair with hyaline-like tissue [20]. Currently, the study is expanded with a follow-up time of 60 months (NCT01626677). CARTISTEM® has recently been introduced in a phase I/II clinical trial in the USA (NCT01733186).

**Table 4:** Clinical studies applying allogeneic mesenchymal stromal cells to a cartilage defect for repair

Cell type	Number of patients	Follow-up	Results	Reference
UMSC	104	48 weeks	Safe application and repair with hyaline tissue	[20] NCT01041001
UMSC	103	60 months		NCT01626677
UMSC	12	24 months		NCT01733186
SMSC				[16,17]
BMMSC with AC	35	1 year	Preliminary: safe application and repair with hyaline tissue	[3,46] NCT02037204

*AC* autologous chondrons, *BMMSC* bone marrow-derived mesenchymal stromal cell, *SMSC* synovium-derived mesenchymal stromal cell, *UMSC* umbilical cord blood-derived mesenchymal stromal cell

A clinical trial using an allograft SMSC-based tissue engineered construct is under review by the Pharmaceutical and Medical Devices Agency of Japan for possible commercialization.

In the Netherlands, we have started an investigator-driven phase I/II clinical trial (IMPACT) using a mixture of rapidly isolated autologous chondrocytes with their pericellular matrix (chondrons) combined with allogeneic BMMSCs in fibrin glue [3,46] (NCT02037204). The inclusion of the targeted 35 patients has recently been completed and no treatment-related adverse events have been observed (the patients are currently at a follow-up ranging from 7 months to 1 year after surgery). Preliminary safety monitoring has not shown any immunological concerns while clinical outcome and structural outcome as measured by MRI and second-look arthroscopies have demonstrated encouraging initial results.

## Induced pluripotent stem cells

The ability to generate iPSCs from somatic cells has created new opportunities for the field of cartilage repair. Just like human embryonic stem cells (hESCs), they show unlimited self-renewal and they can differentiate into all three germ layers (ectoderm, endoderm and mesoderm), but without having the ethical concerns associated with hESCs. However, there are some differences reported in the efficiency to differentiate towards several lineages, such as neural, cardiovascular and hemangioblastic lineages. iPSCs can be generated by overexpressing transcription factors associated with pluripotency, such as Oct3/4, Klf4, c-myc and Sox2. The genetic reprogramming to induce pluripotency is a limiting factor for clinical use as the most efficient viral transductions lead to integration of viral DNA into the chromosome. Reprogramming without causing genetic change has gained recent interest and several non-viral methods using microRNA, synthetic messenger RNA and proteins have been developed.

*In vitro* studies showed chondrogenic differentiation and cartilage formation by iPSCs derived from human fetal neural stem cells [49] and human osteoarthritic chondrocytes [50]. One study showed that overexpression of Oct4 and Klf4 (two-factor reprogramming) was successful in generating iPSCs from murine neural stem cells, which were capable of differentiating into the chondrogenic lineage [51]. Differentiation of iPSCs to the chondrogenic lineage was efficient if they were first differentiated towards an MSC-like intermediate phenotype [52,53].

Chondrogenic cells were also generated directly from somatic cells by reprogramming with c-Myc, Klf4 and the chondrogenic transcription factor Sox9. The cells were non-tumorigenic and had stable karyotypes, and they formed homogeneous hyaline cartilage [54,55].

Diekman and colleagues [56] generated iPSCs from murine fibroblasts and purified the type II collagen-driven green fluorescent protein-expressing cells upon chondrogenic differentiation to obtain a uniformly differentiated cell population. This cell population was subsequently successfully used to fill a defect in an *in vitro* chondral defect model. As it was reported that iPSCs can differentiate easier along the lineages related to the cell type of origin, iPSCs derived from several chondrocyte donors were investigated for their chondrogenic potential [57]. Indeed, these reprogrammed chondrocytes could be differentiated into cartilage-producing chondrocytes more easily than fibroblast-derived iPSCs. However, one of the chondrocyte-derived iPSC lines showed higher aggrecan gene expression level compared with the other generated iPSC cell lines, while no differences were observed in gene expression levels of other chondrogenic markers. So even the chondrogenic potential of iPSCs differs between different iPSC lines.

Although safety precautions and new iPSC generation techniques have been introduced, it remains to be shown that cell fate and phenotype can be controlled without having the risk of teratoma formation. Thus, before preclinical and clinical tests can be done, there is a need for reliable control of the cell fate.

## Ethical considerations in stem cell-based treatments

The design and initiation of clinical trials using stem cells for cartilage repair is ethically challenging [58]. Only a limited number of case reports and clinical trials using a stem cell-based treatment have been reported. Moreover, the end product that is used is often poorly described - critical information on culture methods (if applicable), cell characterization, source, concentration, and carrier are often missing. All these factors have a pronounced influence on the behavior of cells and could, therefore, also affect clinical outcomes of stem cell-based treatments. In the case of BMCs it should be reported how much bone marrow was initially harvested, how much concentrate is used for the treatment and what the CFU/ml is, such as provided by Gobbi and colleagues [35]. The limited number of studies and the lacking information make it hard to accurately predict the risks and clinical outcomes of MSC-based treatments. There are risks associated with the intervention and the harvesting procedures of MSCs, while the invasiveness of both procedures may vary depending on the MSC source and treatment strategy. A risk-benefit ratio should be assessed, as the risk to participants must be proportional to the anticipated benefits. In the relatively new field of MSC-based treatment for cartilage defects, it is hard to predict clinical outcomes and thus benefits for the first individual patients in a clinical study, while the scientific and societal relevance is increased. To be able to assess accurate risk-benefit ratios, negative results should also be published. Moreover, including all data in the European group for Blood and Marrow Transplantation database will enable the risk-benefit assessment for cellular therapy products [59].

Uniform use of outcome parameters facilitates the comparison of treatments used in various clinical studies. There is still an ongoing discussion whether structural cartilage regeneration, clinical improvement or a combination should be the main outcome measure. Clinical improvement is undoubtedly an important outcome measure, but placebo and nonspecific effects can affect the patient’s perspective and it has been suggested that clinical improvement does not necessarily correlate with cartilage tissue regeneration. A second-look arthroscopy and histological evaluation of a biopsy is the golden standard to evaluate structural parameters of cartilage regeneration, but is relatively invasive for patients. A less invasive, but also less detailed and informative, measure is MRI. However, there is only a weak correlation between clinical and MRI outcomes, so the challenge remains to determine how clinical and structural results may correlate [60].

Another important ethical consideration is the selection of an appropriate control group. For a double-blinded randomized controlled trial, the use of a placebo, or in the case of cartilage repair a sham intervention, could be necessary. In the case of MSC-based cartilage repair, the use of a sham group is unacceptable as there is an alternative treatment that provides medical advantage (ACI) and the risks and invasiveness of sham procedures are disproportionate to the social value. ACI can serve as a control. However, it is impossible to compare the two-stage ACI treatment to a single-stage procedure without introducing a sham intervention. It is also unacceptable to test safety, tolerability, pharmacokinetics and pharmacodynamics of MSC-based cell products on healthy volunteers, as the risks and burdens of the intervention are too high.

## Considerations and future perspectives

With respect to the technovolution of articular cartilage repair strategies, it is expected that more single-stage procedures will emerge that utilize a stem cell-based approach as well as procedures using instructive biomaterials that may facilitate the differentiation of MSCs into the chondrogenic lineage. Single-stage cell-based cartilage repair reduces the burden on patients and eliminates a costly cell-expansion phase. As a true one-stage strategy requires only one surgical intervention, additional biopsies apart from the surgery of any kind to isolate chondrocytes or MSCs should be avoided. This suggests that cells should either be isolated during the time frame of one surgery or allogeneic cells should be used.

It is common to select MSCs from a heterogeneous starting population based on their ability to attach and expand on plastic. During culture, they overgrow the other cell types, leading to a culture-expansion-driven isolation of MSCs. For a single-stage strategy this would not be possible if autologous cells were to be used. MSCs could also be isolated by fluorescence-activated cell sorting (FACS) based on their cell surface markers. Antibodies used for the FACS sorting should comply with good manufacturing practice (GMP) regulations for clinical use, which at present is quite expensive. Moreover, as the amount of MSCs is relatively low in adult tissues, it is unlikely sufficient MSCs could be isolated in this way for a single-stage approach. What is more, relatively little information is available on freshly FACS-isolated MSCs with respect to their behavior and chondrogenic capacity. This might differ from expanded MSCs as expansion can favor certain clones. To overcome this problem, autologous bone marrow concentrate (containing the mononuclear cell fraction) and the stromal vascular fraction of adipose tissue are being investigated. Just like the cartilage repair capacities of MSCs from different tissue types are not yet compared in clinical studies, there is no real comparative clinical study on concentrated cell fractions versus MSCs. However, several studies confirmed fibrocartilaginous to hyaline-like repair tissue in cartilage defects treated with BMC [31-37,39,40]. Thus, it might be valuable to investigate the outcomes of concentrated cell fractions compared with expanded MSCs, as allogeneic MSCs are also a viable option for cartilage repair.

Allogeneic MSCs have safely been used in clinical studies. The applicability of allogeneic MSCs opens up the possibility to generate an off-the-shelf cell product for cartilage repair. A clinical grade standardized off-the-shelf product with easy handling for orthopedic surgeons would create a considerable advantage. Critical steps in the development of such a product would be to choose the origin of the cells and the cell carrier, as both factors have a pronounced effect on chondrogenesis and cartilage formation. Besides these factors, such a product should contain cells with proper potency from a single cell line to avoid differences in clinical outcomes due to batch variation. Finally, the production process should be performed in a GMP-licensed cell therapy facility with easy access to the treating hospitals. Although as of yet no preclinical or clinical studies are ongoing which explore the use of iPSCs for cartilage repair, a GMP-grade iPSC cell line might become the basis for such a product in the future, providing that cell fate can be controlled. A hESC cell line would also still possess this therapeutic potential, but would bring along some ethical concerns. Thus far, both autologous MSC-rich concentrates such as BMC and the vascular stromal fraction from adipose tissue, and allogeneic MSCs seem promising cell sources that are currently being used for single-stage treatments of cartilage defects in clinics.

## Conclusion

Implantation of MSCs is a realistic and promising approach for the treatment of cartilage defects, which is increasingly being introduced in early clinical trials. To make optimal use of these different cell types, considerable work remains to be done in terms of finding the optimal cell source, cell dose and carrier along with understanding the (long-term) cell fate and new ethical issues these cell types bring along.

## Note

 This article is part of a thematic series on *Biology and clinical applications of stem cells for autoimmune and musculoskeletal disorders*, edited by Christian Jorgensen and Anthony Hollander. Other articles in this series can be found at http://www.biomedcentral.com/series/MSC.

## Abbreviations

ACI: Autologous chondrocyte implantation
ATMSC: Adipose tissue-derived mesenchymal stromal cell
BMC: Bone marrow concentrate
BMMSC: Bone marrow-derived mesenchymal stromal cell
BMP: Bone morphogenetic protein
CFU-F: Colony-forming unit-fibroblasts
FACS: Fluorescence-activated cell sorting
GMP: Good manufacturing practice
hESC: Human embryonic stem cell
iPSC: Induced pluripotent stem cell
MRI: Magnetic resonance imaging
MSC: Mesenchymal stromal cell
PBMSC: Peripheral blood-derived mesenchymal stromal cell
SMSC: Synovium-derived mesenchymal stromal cell
TGF: Transforming growth factor
UMSC: Umbilical cord blood-derived mesenchymal stromal cell

## References

[1] Gelber AC, Hochberg MC, Mead LA, Wang NY, Wigley FM, Klag MJ (2000). Joint injury in young adults and risk for subsequent knee and hip osteoarthritis. Ann Intern Med.

[2] Brittberg M, Lindahl A, Nilsson A, Ohlsson C, Isaksson O, Peterson L (1994). Treatment of deep cartilage defects in the knee with autologous chondrocyte transplantation. N Engl J Med.

[3] Mastbergen SC, Saris DBF, Lafeber FP (2013). Functional articular cartilage repair: here, near, or is the best approach not yet clear?. Nat Rev Rheumatol.

[4] De Windt TS, Hendriks JA, Zhao X, Vonk LA, Creemers LB, Dhert WJ (2014). Concise review: unraveling stem cell cocultures in regenerative medicine: which cell interactions steer cartilage regeneration and how. Stem Cells Transl Med.

[5] Dominici M, Le Blanc K, Mueller I, Slaper-Cortenbach I, Marini F, Krause D (2006). Minimal criteria for defining multipotent mesenchymal stromal cells. The International Society for Cellular Therapy position statement. Cytotherapy.

[6] Bourin P, Bunnell BA, Casteilla L, Dominici M, Katz AJ, March KL (2013). Stromal cells from the adipose tissue-derived stromal vascular fraction and culture expanded adipose tissue-derived stromal/stem cells: a joint statement of the International Federation for Adipose Therapeutics and Science (IFATS) and the International Society for Cellular Therapy (ISCT). Cytotherapy.

[7] Pittenger MF, Mackay AM, Beck SC, Jaiswal RK, Douglas R, Mosca JD (1999). Multilineage potential of adult human mesenchymal stem cells. Science.

[8] Peng L, Jia Z, Yin X, Zhang X, Liu Y, Chen P (2008). Comparative analysis of mesenchymal stem cells from bone marrow, cartilage, and adipose tissue. Stem Cells Dev.

[9] Huang JI, Kazmi N, Durbhakula MM, Hering TM, Yoo JU, Johnstone B (2005). Chondrogenic potential of progenitor cells derived from human bone marrow and adipose tissue: a patient-matched comparison. J Orthop Res.

[10] Estes BT, Wu AW, Guilak F (2006). Potent induction of chondrocytic differentiation of human adipose-derived adult stem cells by bone morphogenetic protein 6. Arthritis Rheum.

[11] Hennig T, Lorenz H, Thiel A, Goetzke K, Dickut A, Geiger F (2007). Reduced chondrogenic potential of adipose tissue derived stromal cells correlated with an altered TGFbeta receptor and BMP profile and is overcome by BMP-6. J Cell Physiol.

[12] Jones EA, Crawford A, English A, Henshaw K, Mundy J, Corscadden D (2008). Synovial fluid mesenchymal stem cells in healthy and early osteoarthritis: detection and functional evaluation at the single-cell level. Arthritis Rheum.

[13] De Bari C, Dell’Accio F, Tylzanowski P, Luyten FP (2001). Multipotent mesenchymal stem cells from adult human synovial membrane. Arthritis Rheum.

[14] Sakaguchi Y, Sekiya I, Yagishita K, Muneta T (2005). Comparison of human stem cells derived from various mesenchymal tissues: superiority of synovium as a cell source. Arthritis Rheum.

[15] Ando W, Tateishi K, Katakai D, Hart DA, Higuchi C, Nakata K (2008). In vitro generation of a scaffold-free tissue-engineered construct (TEC) derived from human synovial mesenchymal stem cells: biological and mechanical properties and further chondrogenic potential. Tissue Eng Part A.

[16] Ando W, Tateishi K, Hart DA, Katakai D, Tanaka Y, Nakata K (2007). Cartilage repair using an *in vitro* generated scaffold-free tissue-engineered construct derived from porcine synovial mesenchymal stem cells. Biomaterials.

[17] Shimomura K, Ando W, Tateishi K, Nansai R, Fujie H, Hart DA (2010). The influence of skeletal maturity on allogeneic synovial mesenchymal stem cell-based repair of cartilage in a large animal model. Biomaterials.

[18] Chong PP, Selvaratnam L, Abbas AA, Kamarul T (2012). Human peripheral blood derived mesenchymal stem cells demonstrate similar characteristics and chondrogenic differentiation potential to bone marrow derived mesenchymal stem cells. J Orthop Res.

[19] Pievani A, Scagliotti V, Russo FM, Azario I, Ramaldi B, Sacchetti B (2014). Comparative analysis of multilineage properties of mesenchymal stromal cells derived from fetal sources shows an advantage of mesenchymal stromal cells isolated from cord blood in chondrogenic differentiation potential. Cytotherapy.

[20] Cartistem. http://www.medi-post.com/cartistem. Accessed 28 Mar 2015.

[21] Pelttari K, Winter A, Steck E, Goetzke K, Hennig T, Ochs BG (2006). Premature induction of hypertrophy during *in vitro* chondrogenesis of human mesenchymal stem cells correlates with calcification and vascular invasion after ectopic transplantation in SCID mice. Arthritis Rheum.

[22] Harris JD, Siston RA, Brophy RH, Lattermann C, Carey JL, Flanigan DC (2011). Failures, re-operations, and complications after autologous chondrocyte implantation - a systematic review. Osteoarthritis Cartilage.

[23] Dowthwaite GP, Bishop JC, Redman SN, Khan IM, Rooney P, Evans DJ (2004). The surface of articular cartilage contains a progenitor cell population. J Cell Sci.

[24] Williams R, Khan IM, Richardson K, Nelson L, McCarthy HE, Analbelsi T (2010). Identification and clonal characterisation of a progenitor cell sub-population in normal human articular cartilage. PLoS One.

[25] Wakitani S, Mitsuoka T, Nakamura N, Toritsuka Y, Nakamura Y, Horibe S (2004). Autologous bone marrow stromal cell transplantation for repair of full-thickness articular cartilage defects in human patellae: two case reports. Cell Transplant.

[26] Adachi N, Ochi M, Deie M, Ito Y (2005). Transplant of mesenchymal stem cells and hydroxyapatite ceramics to treat severe osteochondral damage after septic arthritis of the knee. J Rheumatol.

[27] Wakitani S, Nawata M, Tensho K, Okabe T, Machida H, Ohgushi H (2007). Repair of articular cartilage defects in the patello-femoral joint with autologous bone marrow mesenchymal cell transplantation: three case reports involving nine defects in five knees. J Tissue Eng Regen Med.

[28] Kuroda R, Ishida K, Matsumoto T, Akisue T, Fujioka H, Mizuno K (2007). Treatment of a full-thickness articular cartilage defect in the femoral condyle of an athlete with autologous bone-marrow stromal cells. Osteoarthritis Cartilage.

[29] Haleem AM, Singergy AA, Sabry D, Atta HM, Rashed LA, Chu CR (2010). The clinical use of human culture-expanded autologous bone marrow mesenchymal stem cells transplanted on platelet-rich fibrin glue in the treatment of articular cartilage defects: a pilot study and preliminary results. Cartilage.

[30] Kasemkijwattana C, Hongeng S, Kesprayura S, Rungsinaporn V, Chaipinyo K, Chansiri K (2011). Autologous bone marrow mesenchymal stem cells implantation for cartilage defects: two cases report. J Med Assoc Thai.

[31] Giannini S, Buda R, Vannini F, Cavallo M, Grigolo B (2009). One-step bone marrow-derived cell transplantation in talar osteochondral lesions. Clin Orthop Relat Res.

[32] Giannini S, Buda R, Battaglia M, Cavallo M, Ruffilli A, Ramponi L (2013). One-step repair in talar osteochondral lesions: 4-year clinical results and t2-mapping capability in outcome prediction. Am J Sports Med.

[33] Buda R, Vannini F, Cavallo M, Grigolo B, Cenacchi A, Giannini S (2010). Osteochondral lesions of the knee: a new one-step repair technique with bone-marrow-derived cells. J Bone Joint Surg Am.

[34] Gigante A, Calcagno S, Cecconi S, Ramazzotti D, Manzotti S, Enea D (2011). Use of collagen scaffold and autologous bone marrow concentrate as a one-step cartilage repair in the knee: histological results of second-look biopsies at 1 year follow-up. Int J Immunopathol Pharmacol.

[35] Gobbi A, Karnatzikos G, Scotti C, Mahajan V, Mazzucco L, Grigolo B (2011). One-step cartilage repair with bone marrow aspirate concentrated cells and collagen matrix in full-thickness knee cartilage lesions: results at 2-year follow-up. Cartilage.

[36] Skowronski J, Skowronski R, Rutka M (2013). Large cartilage lesions of the knee treated with bone marrow concentrate and collagen membrane - results. Ortop Traumatol Rehabil.

[37] First clinical experience with INSTRUCT - a single surgery, autologous cell based technology for cartilage repair. http://www.cellcotec.com/content/P187%20first%20clinical%20experience%20with%20INSTRUCT%20%20final2.pdf.

[38] Nejadnik H, Hui JH, Feng Choong EP, Tai BC, Lee EH (2010). Autologous bone marrow-derived mesenchymal stem cells versus autologous chondrocyte implantation: an observational cohort study. Am J Sports Med.

[39] Giannini S, Buda R, Cavallo M, Ruffilli A, Cenacchi A, Cavallo C (2010). Cartilage repair evolution in post-traumatic osteochondral lesions of the talus: from open field autologous chondrocyte to bone-marrow-derived cells transplantation. Injury.

[40] Skowronski J, Rutka M (2013). Osteochondral lesions of the knee reconstructed with mesenchymal stem cells - results. Ortop Traumatol Rehabil.

[41] Pak J (2011). Regeneration of human bones in hip osteonecrosis and human cartilage in knee osteoarthritis with autologous adipose-tissue-derived stem cells: a case series. J Med Case Rep.

[42] Koh YG, Choi YJ (2012). Infrapatellar fat pad-derived mesenchymal stem cell therapy for knee osteoarthritis. Knee.

[43] Nauta AJ, Fibbe WE (2007). Immunomodulatory properties of mesenchymal stromal cells. Blood.

[44] Ryan AE, Lohan P, O’Flynn L, Treacy O, Chen X, Coleman C (2014). Chondrogenic differentiation increase antidonor immune response to allogeneic mesenchymal stem cell transplantation. Mol Ther.

[45] Li WJ, Chiang H, Kuo TF, Lee HS, Jiang CC, Tuan RS (2009). Evaluation of articular cartilage repair using biodegradable nanofibrous scaffolds in a swine model: a pilot study. J Tissue Eng Regen Med.

[46] Bekkers JE, Tsuchida AI, van Rijen MH, Vonk LA, Dhert WJ, Creemers LB (2013). Single-stage cell-based cartilage regeneration using a combination of chondrons and mesenchymal stromal cells: comparison with microfracture. Am J Sports Med.

[47] Williams SK, Amiel D, Ball ST, Allen RT, Tontz WL, Emmerson BC (2007). Analysis of cartilage tissue on a cellular level in fresh osteochondral allograft retrievals. Am J Sports Med.

[48] HDhollander AA, Verdonk PC, Lambrecht S, Verdonk R, Elewaut D, Verbruggen G (2012). Midterm results of the treatment of cartilage defects in the knee using alginate beads containing human mature allogenic chondrocytes. Am J Sports Med.

[49] Medvedev SP, Grigor’eva EV, Shevchenko AI, Malakhova AA, Dementyeva EV, Shilov AA (2011). Human induced pluripotent stem cells derived from fetal neural stem cells successfully undergo directed differentiation into cartilage. Stem Cells Dev.

[50] Wei Y, Zeng W, Wan R, Wang J, Zhou Q, Qiu S (2012). Chondrogenic differentiation of induced pluripotent stem cells from osteoarthritic chondrocytes in alginate matrix. Eur Cells Mater.

[51] Kuboth S, Kramer J, Rohwedel J (2012). Chondrogenic differentiation *in vitro* of murine two-factor induced pluripotent stem cells is comparable to murine embryonic stem cells. Cells Tissues Organs.

[52] Guzzo RM, Gibson J, Xu RH, Lee FY, Drissi H (2013). Efficient differentiation of human iPSC-derived mesenchymal stem cells to chondroprogenitor cells. J Cellular Biochem.

[53] Koyama N, Miura M, Nakao K, Kondo E, Fuji T (2013). Human induced pluripotent stem cells differentiated into chondrogenic lineage via generation of mesenchymal progenitor cells. Stem Cells Dev.

[54] Hiramatsu K, Sasagawa S, Outani H, Nakagawa K, Yoshikawa H, Tsumaki N (2011). Generation of hyaline cartilaginous tissue from mouse adult dermal fibroblast culture by defined factors. J Clin Invest.

[55] Outani H, Okada M, Yamashita A, Nakagawa K, Yoshikawa H, Tsumaki N (2013). Direct induction of chondrogenic cells from human dermal fibroblast culture by defined factors. PLoS One.

[56] Diekman BO, Christoforou N, Willard VP, Sun H, Sanchez-Adams J, Leong KW (2012). Cartilage tissue engineering using differentiated and purified induced pluripotent stem cells. Proc Natl Acad Sci U S A.

[57] Borestrom C, Simonsson S, Enochson L, Bigdeli N, Brantsing C, Ellerstrom C (2014). Footprint-free human induced pluripotent stem cells from articular cartilage with redifferentiation capacity: a first step towards a clinical-grade cell source. Stem Cells Transl Med.

[58] Niemansburg SL, Teraa M, Hesam H, van Delden JJ, Verhaar MC, Bredenoord AL (2014). Stem cell trials for cardiovascular medicine: ethical rationale. Tissue Eng Part A.

[59] Martin I, Baldomero H, Bocelli-Tyndall C, Passweg J, Saris D, Tyndall A (2012). The survey on cellular and engineered tissue therapies in Europe in 2010. Tissue Eng Part A.

[60] de Windt TS, Welsch GH, Brittberg M, Vonk LA, Marlovits S, Trattnig S (2013). Is magnetic resonance imaging reliable in predicting clinical outcome after articular cartilage repair of the knee? A systematic review and meta-analysis. Am J Sports Med.

